# Energy minimization within target-directed aiming: the mediating influence of the number of movements and target size

**DOI:** 10.1007/s00221-020-05750-w

**Published:** 2020-02-20

**Authors:** James W. Roberts

**Affiliations:** Brain and Behaviour Laboratory, Research Institute of Sport and Exercise Sciences, Liverpool John Moores University, Tom Reilly Building, Byrom Street, Liverpool, L3 5AF UK

**Keywords:** Aiming, Spatial variability, Minimization, Central tendency, Undershooting

## Abstract

In target-directed aiming, performers tend to more greatly undershoot targets when aiming down compared to up because they try to avoid an overshoot error and subsequently minimize the time and energy expenditure that is required to suddenly combat gravitational forces. The present study aims to further examine this principle of time and energy minimization by directly mediating the perceived cost of potential errors as well as the likelihood of their occurrence by manipulating the number of movements and target size, respectively. Participants executed rapid aiming movements in the up/down direction as part of a one-/two-target movement towards a small/large target. Primary movement endpoints showed greater undershooting when aiming in the downward compared to upward direction and small compared to large targets. Meanwhile, the overall movement time showed that slower movements were generated for down compared to up, but only when aiming toward large targets. The failure to mediate the central tendency as a function of the number of movements and target size indicates that the feature of minimization is highly prominent within the performers’ pre-response planning. However, the continued minimization of energy in the presence of large targets may inadvertently cost the movement time.

## Introduction

The trade-off between the movement speed and accuracy within target-directed aiming has long been known (Woodworth 1899; Fitts 1954). That is, the shorter the time it takes to complete the movement, then the greater the chances of missing the target. Alternatively, the more accurately one moves, then the longer it takes to complete the movement. This feature of our sensorimotor system is most problematic given many of our everyday physical interactions are contingent upon being both fast and accurate. Consequently, the trade-off has been explained by the feature of signal-dependent noise (Schmidt et al. 1979; see also, Faisal et al. 2008), where large impulse forces that generate higher accelerations coincide with increase in the trial-by-trial spatial variability (i.e., within-participant standard deviation of movement position)—the quicker the one moves, then the more variable they will be, and thus the more likely they will miss the target.

Because of these inherent features, the motor literature has gladly avoided trying to empirically counter the trade-off and instead focus on ways in which we can manage it. That is, how do we compromise to be sufficiently quick and accurate at the same time? One of the prominent theories surrounding this matter is the optimized submovement model (Meyer et al. 1988). Here, it is suggested that performers strike a compromise by mediating the force of their initial impulse (i.e., primary submovement) in order for their spatial variability to subtend the target boundaries. In the event that the target is initially missed, then performers will concurrently perceive this information via sensory feedback to correct for it, and successfully land on the target (i.e., secondary submovement). In order for this approach to be effective, it was also suggested that the central tendency (i.e., mean) of the primary submovement location will be approximately centre of the target. In the event performers occasionally vary from the mean, then they can provide themselves with an equal chance of reaching slightly under or over the target centre. Alternatively, biasing the central tendency to one side of the target will see a larger portion of the distribution reaching outside the target boundaries and thus an increased likelihood of missing the target.

More recently, the time and energy minimization model has been forwarded (Elliott et al. 2004; see also, Elliott et al. 2017), which predominantly agrees with the tenets of the optimized submovement model (e.g., mediating forces, secondary submovement correction). However, it alternatively states that the central tendency of the primary submovement location should come under the target, at least when the spatial variability is deemed to likely exceed the target boundaries (see also, Worringham 1991). This feature holds that performers anticipate the likelihood of requiring a secondary submovement correction. At this juncture, it is deemed that not all errors (undershoot vs. overshoot) are equal as overshoots require more time and energy to correct to overcome inertia and reverse the limb onto the target. Thus, performers can positively avoid this unfavourable situation by initially undershooting the target and correcting the movement in a direction that is consistent with the primary submovement. While an undershoot error still requires a secondary submovement correction, it is deemed to be optimal given this form of correction (re-acceleration/discontinuities) takes less time and energy compared to the type of correction (reversal) required following an overshoot (for time minimization effects following undershoots vs. overshoots; see Elliott et al. 2004; Roberts et al. 2018).

The one aspect of this model that has been closely examined includes the minimization of energy expenditure. For example, having performers aim up and down within the vertical axis is suggested to mediate the cost of potential errors to energy expenditure following the primary submovement (Lyons et al. 2006). To elucidate, the downward direction of aiming movements assumes a greater cost of an overshoot because a subsequent reversal requires more energy to work against gravitational forces. Alternatively, the upward direction assumes an initial overshoot that requires a reversal may instead utilise gravitational forces to land on the target. As a result, performers tend to more greatly undershoot (see Bennett et al. 2012; Burkitt et al. 2017; Roberts and Grierson 2019), and subsequently may take a longer time and displacement to correct the movement (Elliott et al. 2014; Roberts et al. 2016a, b), when aiming down compared to up.

Subsequent attempts have been made to overturn this central tendency by issuing a further requirement to move towards a second target (i.e., two-target movements) (Roberts et al. 2016a, b). Indeed, it was suggested that the perceived advantage of undershooting when aiming down compared to up may no longer apply when there is a second movement being prepared. Thus, all of a sudden, there should be a limited difference in the central tendency during the first- of a two-target movement (e.g., down–down, down–up, up–up, up–down). However, there appeared a continued tendency to more greatly undershoot when aiming down compared to up. This outcome was suggested to result from the task parameters inadvertently causing performers to integrate the two movements and consequently control them as a unitary sequence (e.g., Adam et al. 2000; see also, Bested et al. 2018; Khan et al. 2010). Thus, the preference to minimize the time and energy expenditure of potential errors within the second movement may have contaminated the central tendency of the first movement. The present study aims to adapt this principle as an examination of the minimization model by more clearly separating the fore mentioned two movements when aiming in the up and down directions.

On a separate—but not mutually exclusive—note, is the influence of target size. Inherent within the minimization model and its inverse relation between primary movement endpoint and spatial variability is the fact that the enhanced tendency to undershoot is strongly determined by the anticipated likelihood of missing the target. That is, if the spatial variability is deemed to likely miss the target, then there is an increased likelihood of needing to correct the movement, which is best coming from under the target. Alternatively, if the spatial variability is strongly perceived as subtending the target boundaries, then there is less likely a need to correct the movement and thus we can immediately reach to the target centre. At this juncture, it stands to reason that there will be an increased tendency to undershoot when faced with a “small” compared to “large” target, where there will be a respective decreased chance of the primary submovement reaching inside the target. Along these lines, previous research has shown the opposing set of findings, where there is a central tendency at target centre for the small targets, and increased undershooting for the large targets (Slifkin and Eder 2017). These findings were leveraged as greater support for the optimized submovement model, where there is a central tendency located at target centre by default. On the other hand, performers may undershoot when aiming to supremely large targets (~ 8 cm) to minimize the overall movement amplitude as opposed to the cost of a potential error per se. Notably, these findings involved a video-based translational setup (i.e., virtual representation of digitized movements) that analysed the very end of the movement. Alternatively, the present study additionally aims to explore the influence of target size within a real-world three-dimensional setup that primarily isolates the primary submovement (of two-component movements).

The present study had participants execute target-directed aiming movements in the up and down direction to either one or two targets. The two-target conditions involved continuing within the same (extension), or reversing to the opposing (reversal), direction of the first movement. Of interest, the participants were made fully aware of the required number of movements courtesy of a pre-cue before each trial began. Meanwhile, the two-target movements were positively brokered into individual movements as opposed to a unitary sequence by introducing a prolonged foreperiod following the first movement (1300–2800 ms). In addition, the influence of target size was observed by incorporating either small (0.5 cm) or large (2 cm) targets that would mediate the proximity of the participants’ spatial variability with respect to the target boundaries.

It was hypothesised that there would be an increased tendency to undershoot the primary movement at the first target when aiming down compared to up during the one-target movements. However, there would be no systematic difference between the movement directions during the two-target movements (as indicated by direction × sequence interaction). That is, the requirement of a second movement in an extension or reversal of the first movement may dictate a limited cost of potential errors at the first target because participants are conscious of the fact that there is an additional second movement. Meanwhile, it was predicted that there would be an increased tendency to undershoot when moving down compared to up for the small target. However, the difference between the movement directions will no longer unfold for the large target (as indicated by a direction × size interaction). This outcome is adapted from the principle that the more closely spatial variability can subtend the target boundaries, then the lesser the concern for the perceived cost of potential errors.

## Method

### Participants

Sixteen participants (age range = 20–28 years; male = 11; females = 5; self-report right-handed = 15) agreed to take part in the study. No participants reported any sensory-motor or neurological disorder. The study was approved by the local ethics committee, designed and conducted in accordance with the Declaration of Helsinki (2013).

### Apparatus and task

**Fig. 1:**
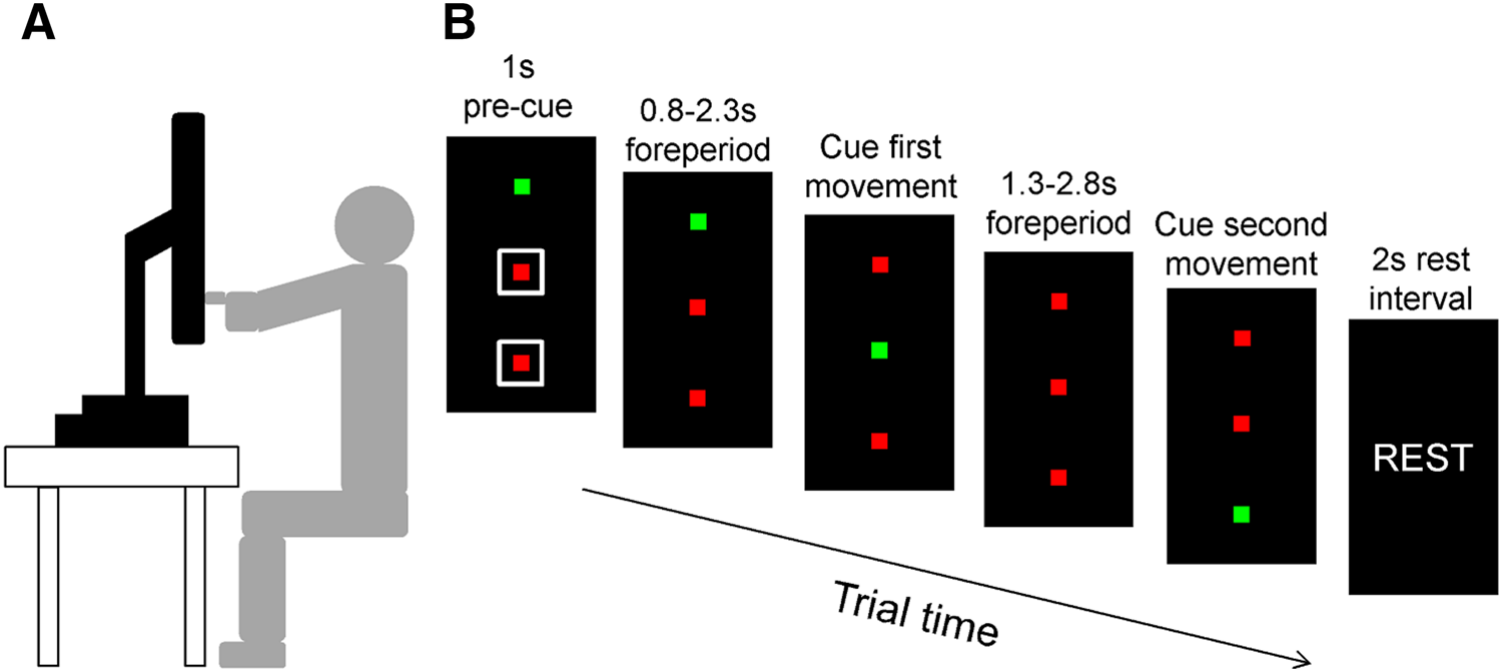
**a** Experimental setup including display (black), table top (white) and participant seating position (grey). **b** Time course of a single trial (illustration depicts a two-target extension movement)

Stimuli were presented on an LCD monitor (47.5 cm × 27.0 cm; temporal resolution = 75 Hz; spatial resolution = 1920 × 1080 pixels) that was covered by a 2-mm acrylic sheet and mounted on a computer unit placed on top of a table. The monitor was rotated 90° around its arm in order for the long edge to appear in the vertical axis. The height of the monitor was adjusted so that the centre would appear at approximate eye level of the participant (see Fig. 1a). Participants wore a glove with a NO/NC button micro-switch (Saia-Burgess Electronics, Murten, Switzerland) attached at the tip of the index finger, which was connected to the serial port of the computer. In addition, a retro-reflective marker was fitted on top of the index finger of the glove and thus appeared orthogonal to the orientation of the micro-switch. A Vicon camera system (Vicon Vantage, 16-megapixel resolution) was used to capture the entire trajectory of the marker at a 200 Hz sampling rate.

Stimuli were both generated and controlled via Matlab (The Mathworks Inc., Natick, MA) running Psychtoolbox-3 (Pelli 1997). There were three small (5 × 5 mm) or large (20 × 20 mm) squares presented along the vertical axis on the monitor and separated by 160 mm (centre-to-centre). The task required participants to initially make contact with one of the squares on the screen using the micro-switch that was attached to the index finger of their dominant limb. Therein, participants would commence their movement by releasing the micro-switch from the screen. They were required to move as quickly and accurately as possible and end their movement on the target by once more bringing the micro-switch in contact with the screen.

### Procedure

Figure 1b indicates the separate events that unfolded within each trial. Stimuli were first presented with two of the squares being highlighted in red, while one of the top/bottom squares were highlighted in green to indicate where the movement should start. All the squares were made either small (5 mm) or large (20 mm) depending on the trial condition, and represented the home and target locations for the main aiming task. A trial would commence as soon as participants made contact with the green square using the micro-switch. A white unfilled square (30 × 30 mm) appeared immediately afterward around one or two of the squares for a period of 1 s to indicate the direction and number of target movements that were required to be executed. The first movement of each trial was to be always executed towards the centre square location with the possibility of ending (one movement), or continuing the movement in the same (extension movement) or opposing (reversal movement) direction. To ensure participants recognised the pre-cues, they were instructed to verbalise how many target movements they were required to execute (e.g., “ONE” or “TWO”). Following a random foreperiod (800–2300 ms), the first centre square location changed from red to green, which signalled the participant to move to the first target. If there was a requirement to execute a further movement, then participants had to hold on for the next onset cue. Following another random foreperiod (1300–2800 ms), the second top/bottom square location was changed from red to green, which signalled the participant to move to the second target. Once all the required movements were completed, then a prompt (“REST”) was issued on the screen for a period of 2 s to remind participants that they should bring their arm down and rest it on the table top.

The study conditions (*n* = 12) were categorised by the direction of the first movement, size of the targets and presence of a second movement requirement. A random permutation of the conditions was repeated over 10 trials, which accumulated to a total of 120 trials. Notably, there were 80 trials requiring a second movement, which effectively equated to 200 individual movements (120 + 80 trials).

### Data management and analysis

Position time series data from the primary direction (*z*-axis) of the index finger marker was selected for further processing and analysis. The position data were single, double and triple differentiated to obtain instantaneous velocity, acceleration and jerk, respectively. Individual trajectories were plotted and observed with a view to manually selecting the range of data featuring the primary task movement courtesy of a graphical user interface. Movement onset was marked by parsing backward from peak velocity until the first sample that reached < 10 mm/s. Movement offset was marked by parsing forward from peak velocity until the first sample reached < 10 mm/s for a temporal window of > 40 ms (eight samples). To isolate the presence a primary submovement, the movements were further parsed from peak deceleration until one of the following criteria was met: (i) positive-to-negative zero-crossing in velocity (type 1; reversal); (ii) negative-to-positive zero-crossing in acceleration following peak deceleration (type 2; re-acceleration); (iii) positive-to-negative zero-crossing in jerk (type 3; discontinuities) (Elliott et al. 2014; Fradet 2008). In the event that more than one criteria were met, then the one earliest in time from the very start of the movement was selected. The sign ( ±) associated with the above criteria was switched to undertake the same parsing algorithm for the downward aiming movements.

First, the reaction time in preparation for the first movement was calculated by subtracting the time at the start of the movement from the time at target onset. Therein, the key dependent measures involved the location and time of the primary movement and terminal movement endpoints. This procedure involved calculating the difference in location between the limb and target centre, which means negative scores indicate target undershooting and positive scores indicate target overshooting. Meanwhile, the dispersion of the movements was represented by calculating the population standard deviation of the fore mentioned error scores. Finally, the movement time was calculated by simply subtracting the time at the start of the movement from the time at the primary or terminal movement endpoints.

Trials were the reaction times were < 100 ms, movement times were > 1000 ms, and absolute constant error scores were > 12.5 mm from the target boundary (small target: > 15 mm; large target: 22.5 > mm) were removed prior to the analysis. Mean participant values at the first movement were forwarded to a two-way repeated-measures ANOVA: 2 directions (up, down), 2 sizes (small, large), and 3 sequences (one, extension, reversal). In the event of a violation in the assumption of sphericity (courtesy of Mauchly’s test), the Huynh–Feldt corrected value was adopted provided epsilon was > 0.75. If otherwise, then the Greenhouse–Geisser corrected value was adopted. Partial eta-squared (*ƞ*^*2*^) indicated the size of any treatment effects. Significant effects featuring more than two means were decomposed by the Tukey HSD post hoc procedure. Statistically significant effects were declared when *p* < 0.05.

## Results

Reaction times in preparation for the first movement revealed a significant main effect of size, *F*(1, 15) = 36.73, *p* < 0.05, *partial ƞ*^*2*^ = 0.71, which indicated a longer time to initiate movements when presented with small compared to large targets (see Fitts and Peterson 1964). There was also a significant main effect of sequence, *F*(2, 30) = 9.94, *p* < 0.05, *partial ƞ*^*2*^ = 0.40, which indicated a significantly longer time for the extension compared to one-target and reversal movements, and no significant difference between the one-target and reversal movements. Meanwhile, there was no significant main effect of direction, *F*(1, 15) = 2.25, *p* > 0.05, *partial ƞ*^*2*^ = 0.13, nor any statistically significant interactions (*Fs* < 1).

### Primary movement

**Fig. 2:**
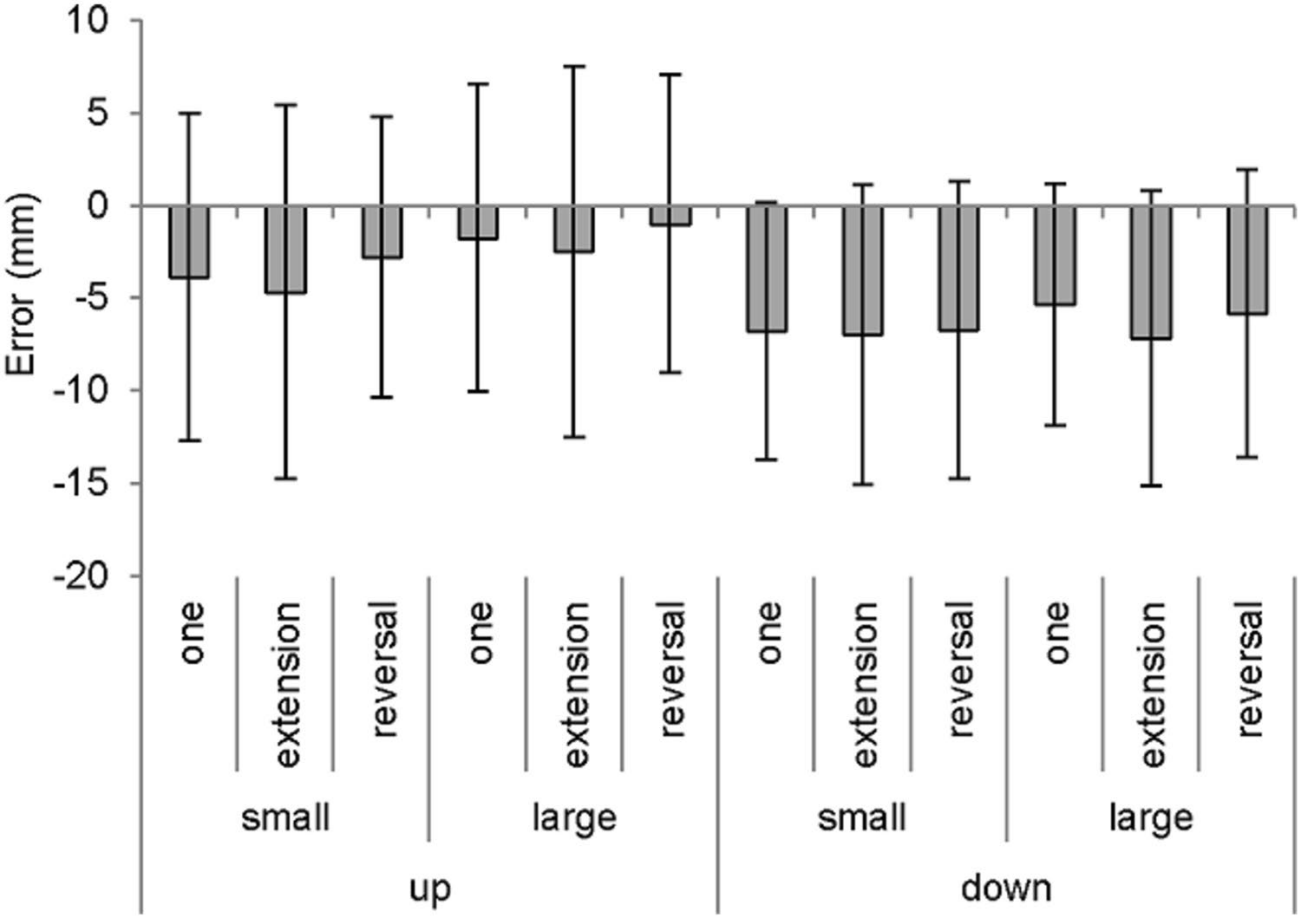
Mean spatial tendency of the primary movement endpoint as a function of direction, size and sequence. Error bars indicate the mean spatial dispersion (within-participant variability)

For spatial tendency, there was a significant main effect of direction, *F*(1, 15) = 4.64, *p* < 0.05, *partial ƞ*^*2*^ = 0.24, size, *F*(1, 15) = 12.79, *p* < 0.05, *partial ƞ*^*2*^ = 0.46, and sequence, *F*(2, 30) = 3.37, *p* < 0.05, *partial ƞ*^*2*^ = 0.18, which indicated greater undershooting for down compared to up, small compared to large, and extension compared to reversal movements (*p* < 0.05) (see Fig. 2). However, there were no significant interactions (direction *x* size: *F*(1, 15) = 1.16, *p* > 0.05, *partial ƞ*^*2*^ = 0.07; remaining statistical interaction: *Fs* < 1). For spatial dispersion, there was no significant main effect of direction, *F*(1, 15) = 2.28, *p* > 0.05, *partial ƞ*^*2*^ = 0.13, nor size, *F*(1, 15) = 0.15, *p* > 0.05, *partial ƞ*^*2*^ = 0.01. However, there was a significant main effect of sequence, *F*(2, 30) = 4.77, *p* < 0.05, *partial ƞ*^*2*^ = 0.24, which indicated significantly more variability for extension movements compared to one-target movements (*p* < 0.05) with a similar trend compared to reversal movements (*p* > 0.05). There were no significant interactions (direction × sequence: *F*(2,30) = 2.77, *p* > 0.05, *partial ƞ*^*2*^ = 0.16; remaining statistical interactions: *Fs* < 1). For time at the primary movement endpoint, there was no significant main effect of direction, nor sequence, *Fs* < *1*, although there was for size, *F*(1, 15) = 6.52, *p* < 0.05, *partial ƞ*^*2*^ = 0.30, as time was extended in the presence of small compared to large targets (see Table 1).

**Table 1:** Mean (± SE) time at the primary movement endpoint (ms) as a function of direction, size and sequence

	Small			Large		
	One	Extension	Reversal	One	Extension	Reversal
Up	417 (19)	424 (17)	430 (19)	417 (18)	411 (17)	421 (20)
Down	425 (18)	430 (19)	423 (17)	423 (19)	420 (18)	421 (16)

### Movement end

**Fig. 3:**
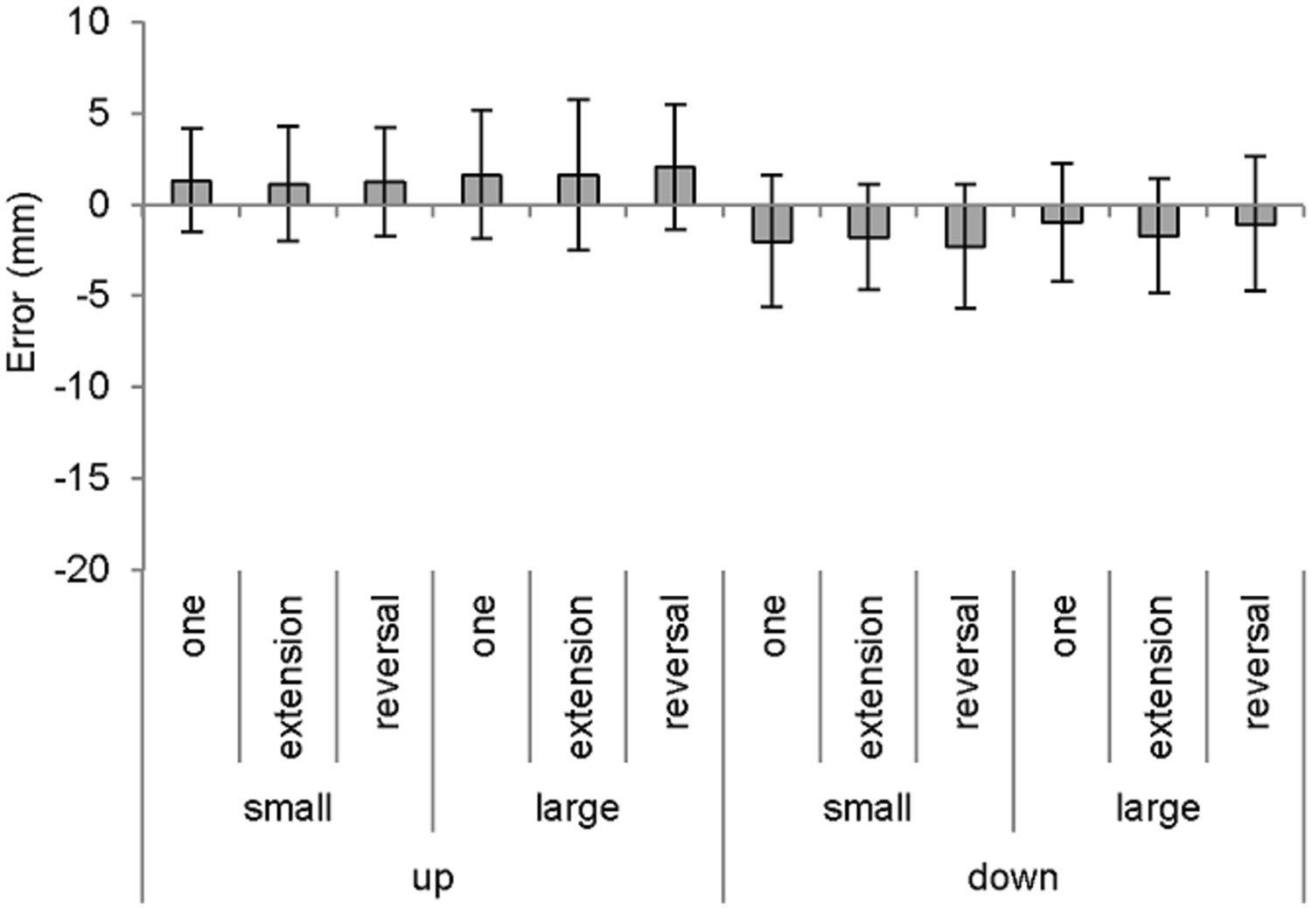
Mean constant error of the terminal movement endpoint as a function of direction, size and sequence. Error bars indicate the mean variable error (within-participant variability)

For constant error, there was a significant main effect of direction, *F*(1, 15) = 4.89, *p* < 0.05, *partial ƞ*^*2*^ = 0.25, and size, *F*(1, 15) = 7.31, *p* < 0.05, *partial ƞ*^*2*^ = 0.33, which indicated a greater undershoot following down compared to up, and small compared to large (see Fig. 3). However, there was no significant main effect of sequence, *F*(2,30) < 1. What’s more, there were no significant interactions (size × sequence: *F*(2, 30) = 1.64, *p* > 0.05, *partial ƞ*^*2*^ = 0.10; direction × size × sequence: *F*(2, 30) = 1.04, *p* > 0.05, *partial ƞ*^*2*^ = 0.07; remaining statistical interactions: *Fs* > 1). For variable error, there was a significant main effect of size, *F*(1, 15) = 13.33, *p* < 0.05, *partial ƞ*^*2*^ = 0.47, which indicated lower variability for small compared to large targets. There was no significant main effect of direction, *F*(1, 15) = 3.89, *p* = 0.07, *partial ƞ*^*2*^ = 0.21, nor sequence, *F*(2, 30) = 1.43, *p* > 0.05, *partial ƞ*^*2*^ = 0.09. What’s more, there were no significant interactions (direction × size: *F*(1, 15) = 1.51, *p* > 0.05, *partial ƞ*^*2*^ = 0.09; direction × size × sequence: *F*(2, 30) = 2.50, *p* > 0.05, *partial ƞ*^*2*^ = 0.14; remaining statistical interactions: *Fs* < 1). For overall movement time, there was a significant main effect of size, *F*(1, 15) = 13.92, *p* < 0.05, *partial ƞ*^*2*^ = 0.48, as time was extended in the presence of small compared to large targets (see Table 2). Meanwhile, there was no significant main effect direction, nor sequence, *Fs* < 1. Most importantly, there was a significant direction × size interaction, *F*(1, 15) = 4.68, *p* < 0.05, *partial ƞ*^*2*^ = 0.24, which indicated a significantly shorter time for up compared to down when in the presence of large targets (*p* < 0.05), although there was no significant difference in the presence of small targets (*p* > 0.05). There were no further significant interactions (direction × sequence: *F*(2, 30) = 3.07, *p* = 0.061, *partial ƞ*^*2*^ = 0.17; remaining statistical interactions: *Fs* < 1).

**Table 2:** Mean (± SE) overall movement time at the terminal movement endpoint (ms) as a function of direction, size and sequence

	Small			Large		
	One	Extension	Reversal	One	Extension	Reversal
Up	489 (24)	492 (23)	497 (24)	465 (22)	469 (21)	473 (23)
Down	500 (23)	497 (23)	491 (21)	484 (24)	489 (22)	481 (20)

## Discussion

The minimization model states that the trade-off between speed and accuracy, and the associated signal-dependent noise (Schmidt et al. 1979; see also, Faisal et al. 2008), is partially managed by initially undershooting the primary movement with a view to undertaking a less time- and energy-consuming secondary submovement correction (Elliott et al. 2004; see Elliott et al. 2017). This model has been heavily substantiated by the enhanced tendency to undershoot when aiming down compared to up to avoid the cost of a potential overshoot error that requires a correction against gravitational forces (Lyons et al. 2006; Roberts et al. 2016a, b). The following study aimed to manipulate this central tendency by mediating factors that precisely influence minimization. That is, participants executed aiming movements in the up and down directions as part of a one- or two-target movement that featured small or large targets. It was predicted that the typical finding of an enhanced undershoot when aiming down compared to up would solely manifest within trials that involved only one-target movements and small target sizes. The findings revealed that there was a significant undershoot bias for the downward compared to upward direction, and small- compared to large-sized targets. These differences unfolded independently of the other experimental factors, as there were no statistically significant interactions. However, there was an extended movement time for aiming movements in the downward compared to upward direction, but only when aiming to the large target.

The enhanced undershooting when aiming down compared to up is consistent with previous evidence, which indicates how performers adapt their pre-planned primary movement in accordance with anticipated cost of potential errors (Lyons et al. 2006). To elucidate, the cost of having to reverse the limb following an overshoot error becomes even greater when having to move against gravitational forces. Thus, the difference in the extent of undershooting is deemed an adaptive response that positively avoids the occurrence of such a costly error. Of interest, the central tendency of primary movement failed to be mediated by the pre-cued number of movements, where performers were potentially instructed to prepare a second extension or reversal movement. Thus, it appears the energy-minimizing effects of undershooting in the downward compared to upward direction unfolded regardless of the upcoming movement demands (see Roberts et al. 2016a, b). Indeed, if performers were primarily concerned with the cost of potential errors within the broader context of preparing a second movement, then we should have observed limited differences between the up and down directions during the two-target movements. In other words, it appears the cost of potential errors primarily holds an influence within-trials (i.e., trial *n*), as opposed to between-trials (i.e., *trialn* + 1). This principle contrasts with the findings from open(no vision)- vs. closed(vision)-loop control, where the utilisation of visual sensory feedback and the subsequent precision of movements are directly influenced by events that are accumulated from other trials (i.e., trial *n*-1) (Burkitt et al. 2015; Cheng et al. 2008; see also, Whitwell et al. 2008). Taken together, it could be argued that aiming movements are prepared purely in the context of the immediate costs regardless of the upcoming trial demands, although the previously evidenced utilisation of feedback for corrections is contingent upon previous experience (see also, Proteau et al. 1987).

At this juncture, it is perhaps worthwhile evaluating the impact of two-target movements on the overall preparation and execution of the first movement. The present study attempted to overtly avoid the profound integration that takes place for continuous sequential movements (e.g., Roberts et al. 2016a, b) by prolonging the time between the first and second movements (1300–2800 ms foreperiod). With this in mind, an analysis of the initial reaction times showed at least some influence of having to execute a second movement as the extension movements took longer to initiate compared to both the one-target and reversal movements. However, these findings are not fully compatible with the ubiquitous notion of an extended reaction time for multi- compared to one-target movements (Henry and Rogers 1960; Khan et al. 2006; Klapp 1995). Indeed, the extended time for the two-target movements in this study appeared to be isolated to the extension movements. While beyond the scope and primary interest of the present study, it can only be speculated that this extended time to initiate extension movements was also related to their enhanced spatial variability and subsequently greater undershooting.

Similar to the effects of direction on target undershooting, the enhanced undershooting to small compared to large targets also reflects the anticipated cost of potential errors. To elucidate, the increased need to control the movement towards small compared large targets courtesy of the higher precision demands may benefit from the primary submovement being located under the target. This suggestion is also consistent with the finding that the increased undershooting during extension trials coincided with increases in variable error—the more variable and less precise the performer, then the greater the tendency to undershoot (see Worringham 1991).

Of interest, the previously mentioned undershoot bias for the downward compared to upward direction failed to be differentiated as a function of target size. Indeed, if performers factored in the cost of potential errors, as well as the likelihood of initially missing the target, then it stands to reason that the large target would elicit less of a difference between the movement directions. This prediction is adapted from the notion that increase in the target size can allow the spatial variability to more closely subtend the target boundaries. While the spatial variability continued to marginally exceed the large target boundaries (mean range of effective target width = 26.98–33.36 mm; see also Fig. 2), it is clear that the proximity between the spatial variability and target boundaries was substantially decreased during trials with the large target. Thus, the enhanced undershooting for the downward compared to upward direction appears to indicate a more conservative approach than perhaps originally thought, where performers are either insensitive or require further recourse to justify less undershooting (extending the primary movement amplitude) while in the presence of larger-sized targets.

On reflection of the movement times, this approach could become suboptimal as there was a longer time generated for the downward compared to upward direction, but only when there was a large target. Presumably, the enhanced undershooting in the downward direction coincided with a longer time and displacement to undertake the secondary submovement correction. However, the upward direction reached further into the distance, and thus required a shorter time and displacement to complete the secondary submovement, which in many instances appeared non-functional given they unfolded inside the target (see Fig. 2).

In conclusion, the present findings strongly support the principle of time and energy minimization, along with the associated evidence of increased undershooting when aiming down compared to up. This finding has been leveraged as support for the notion that not all errors are equal as more time and energy is required to correct overshoots. Of interest, the present study showed how this feature of the sensorimotor system unfolds regardless of any upcoming movement requirements as performers adopt an approach that primarily minimizes the costs within the present movement. That said, the study presents early evidence that the robust attempts to minimize energy expenditure could come at the expense of time whenever there are minimal demands on accuracy (i.e., larger target).
